# Retinal abnormalities, although relatively common in sleep clinic patients referred for polysomnography, are largely unrelated to sleep-disordered breathing

**DOI:** 10.1007/s11325-022-02679-y

**Published:** 2022-07-08

**Authors:** Terence C. Amis, Rita Perri, Sharon Lee, Meredith Wickens, Gerald Liew, Paul Mitchell, Kristina Kairaitis, John R. Wheatley

**Affiliations:** 1 Ludwig Engel Centre for Respiratory Research, The Westmead Institute for Medical Research, The University of Sydney, Sydney, NSW Australia; 2 Westmead Clinical School, University of Sydney, Westmead, NSW Australia; 3 Department of Respiratory and Sleep Medicine, Westmead Hospital, Sydney, NSW Australia; 4 Department of Ophthalmology, Westmead Hospital, Sydney, NSW Australia; 5 Centre for Vision Research, The Westmead Institute for Medical Research, The University of Sydney, Sydney, NSW Australia

**Keywords:** Obstructive sleep apnea, Sleep-disordered breathing, Retinal photography, Peripapillary atrophy, Age-related macular degeneration

## Abstract

**Study objectives.:**

There has been long-standing interest in potential links between obstructive sleep apnea (OSA) and eye disease. This study used retinal photography to identify undiagnosed retinal abnormalities in a cohort of sleep clinic patients referred for polysomnography (PSG) and then determined associations with PSG-quantified sleep-disordered breathing (SDB) severity.

**Methods:**

Retinal photographs (*n* = 396 patients) were taken of each eye prior to polysomnography and graded according to validated, standardized, grading scales. SDB was quantified via in-laboratory polysomnography (PSG; *n* = 385) using standard metrics. A questionnaire (*n* = 259) documented patient-identified pre-existing eye disease. Within-group prevalence rates were calculated on a per patient basis. Data were analyzed using multivariate logistic regression models to determine independent predictors for retinal abnormalities. *P* < 0.05 was considered significant.

**Results:**

Main findings were (1) 76% of patients reported no pre-existing “eye problems”; (2) however, 93% of patients had at least one undiagnosed retinal photograph-identified abnormality; (3) most common abnormalities were drusen (72%) and peripapillary atrophy (PPA; 47%); (4) age was the most common risk factor; (5) diabetes history was an expected risk factor for retinopathy; (6) patients with very severe levels of SDB (apnea hypopnea index ≥ 50 events/h) were nearly three times more likely to have PPA.

**Conclusion:**

Retinal photography in sleep clinic settings will likely detect a range of undiagnosed retinal abnormalities, most related to patient demographics and comorbidities and, except for PPA, not associated with SDB. PPA may be indicative of glaucoma, and any association with severe SDB should be confirmed in larger prospective studies.

**Supplementary Information:**

The online version contains supplementary material available at 10.1007/s11325-022-02679-y.

## Introduction

Population screening for undiagnosed retinal disease using fundus photography is now deployed worldwide in a variety of clinical and community settings. The now ubiquitous nature of retinal photographic capabilities, combined with new technologies (e.g., smart phone fundus photography, telemedicine, and artificial intelligence [1]), raises the question of whether retinal photography screening could be instituted more widely in non-ophthalmology medical clinic settings, particularly those attended by potentially at-risk patient cohorts, such as sleep clinics.

Obstructive sleep apnea is a common condition, with a prevalence of 13 to 33% in men and 6 to 19% in women, although this may be substantially higher in older age groups [2]. Given the pathophysiological features of OSA, including sleep fragmentation, increased arousals, sympathetic activation, nocturnal blood pressure surges, and intermittent hypoxia, recent ophthalmology reviews have nominated putative pathways via which the physiology of the retina may be at risk of disruption from sleep-disordered breathing (SDB) events [3, 4].

Over the last three decades, there has been developing interest in exploring links between OSA and eye diseases. One of the earliest described associations was with floppy eyelid syndrome [5]. Since then, associations have been proposed for glaucoma [6], non-arteritic anterior ischemic optic neuropathy (NAION) [7], retinal vein occlusion [8], and central serous chorioretinopathy [9]. OSA has also been reported as an independent risk factor in the progression of diabetic retinopathy [10] and has been linked to non-responsiveness to intra-vitreal anti-vascular endothelial growth factor (anti-VEGF) therapy for exudative AMD [11]. Diabetic retinopathy and AMD are of particular interest in sleep clinic cohorts because of shared risk factors of older age and obesity.

As part of a study exploring links between OSA and retinal microvascular morphology, we had previously obtained retinal photographs together with overnight, in-laboratory, polysomnography (PSG) data in patients referred to a sleep clinic for investigation of SDB. For clinical oversight reasons, all retinal photographs were reviewed for the presence of retinal abnormalities (separate to the microvascular morphology). In the present manuscript, we report these findings and test for any associations with SBD severity.

## Methods

This cross-sectional, observational study was approved by the Western Sydney Local Health District Human Research Ethics Committee (local reference number HREC/11/WMEAD/296).

### Study participants

Retinal pathology reports were available for 396 patients, consisting of 264 non-diabetics included in the retinal microvascular morphology study, and an additional 132 patients not included in that study. All of these patients had been referred to a sleep clinic for assessment of their SDB status, and their prior retinal abnormality status was unknown. PSG was performed and the results reported for 385 patients.

### Study protocol

The research records were reviewed, and the following data extracted:

### Anthropometric and medical history data

The anthropometric and medical history data are as follows: height, weight, body mass index (BMI), waist circumference, hip circumference, waist-hip ratio (WHR), neck circumference, blood pressure, detailed medical history (history of hypertension, hypercholesterolemia, and diabetes), and smoking history (current or past). Patients self-identified ethnicity in response to an in-house questionnaire. For analysis, these data were collapsed into 2 categories: Caucasian and Non-Caucasian.

### Eye problems questionnaire

Patients were asked if they had any eye problems and to detail the nature of those problems. These data were available for 259 patients.

### Polysomnography

SDB was assessed by standard in-laboratory, overnight polysomnography (PSG). Studies were scored, using Compumedics Profusion PSG3 software (Compumedics Limited; Abbotsford, Victoria, Australia) according to standard guidelines [12]. The following metrics were extracted from the sleep study report: apnea–hypopnea index (AHI), respiratory disturbance index (RDI), oxygen desaturation index (ODI > 3%), arousal index (AI), and the percentage of sleep time spent with oxygen saturation below 90% (SpO_2_ < 90%). For hypopneas, oxygen desaturation ≥ 3% was required. The RDI was defined as the sum of apneas, hypopneas, and respiratory effort–related arousals (RERA) per hour.

### Retinal photography

For most patients, retinal photographs were obtained on the evening they attended the Westmead Hospital Clinical Sleep Laboratory for overnight polysomnography. For a minority, retinal photography occurred at clinical consultation or on the morning after polysomnography. Retinal images were obtained for each eye using a non-mydriatic digital fundus camera (Canon CR2-Plus, Canon, Japan) either during the initial clinic visit (morning; 99 participants) or on the evening the patient underwent overnight PSG (297 participants). Pupil dilation (one drop of 1% Tropicamide per eye) was used for 265 patients. Three retinal images were obtained for each eye: (1) centered on the optic disc; (2) centered on the macula; and (3) temporal but including the fovea.

### Retinal pathology assessment

A single, experienced, fully trained, ophthalmology technician reviewed all images and reported the presence of retinal abnormalities identified according to published criteria and classification systems [13]. AMD lesions were classified using the criteria proposed by Ferris et al. [14]. Diabetic retinopathy was scored according to the modified Early Treatment of Diabetic Retinopathy grading scale [13]. Where the technician was uncertain in classifying an abnormality, or if the abnormality was considered to be of potential clinical significance, the image was reviewed by an ophthalmologist (PM) who determined the appropriate classification and directed clinical care where required.

### Data analysis

All statistical analyses were performed using SPSS version 24.0 (IBM SPSS Statistics for Windows, Version 24.0. Armonk, NY: IBM Corp.). *P* < 0.05 was considered significant.

For both questionnaire data and retinal-photograph-identified abnormalities, we calculated within-group prevalence rates on a per patient basis. Patients were deemed to have a specific retinal-photograph-identified abnormality if it was reported in at least one eye. Anthropometric data were expressed as mean (± standard deviation) or median (interquartile range), where appropriate, for continuous variables, and frequency and percentage for categorical variables.

Logistic regression (backwards step) was used to test whether any SBD severity metrics constituted a significant risk factor for specific retinal abnormalities. SDB severity metrics are well known to be highly skewed and collinear. Therefore, these data were first log transformed (after adding 1 to deal with potential zero values where required) and then tested separately. We also tested for severe (AHI: ≥ 30 events/h) and very severe (AHI: ≥ 50 events/h) SDB as categorical variables. Logistic regression models were adjusted for age, plus other known abnormality-specific risk factors as described in the literature and where that information was available in our data set (e.g., smoking history, diabetic status, ethnicity).

## Results

**Fig. 1:**
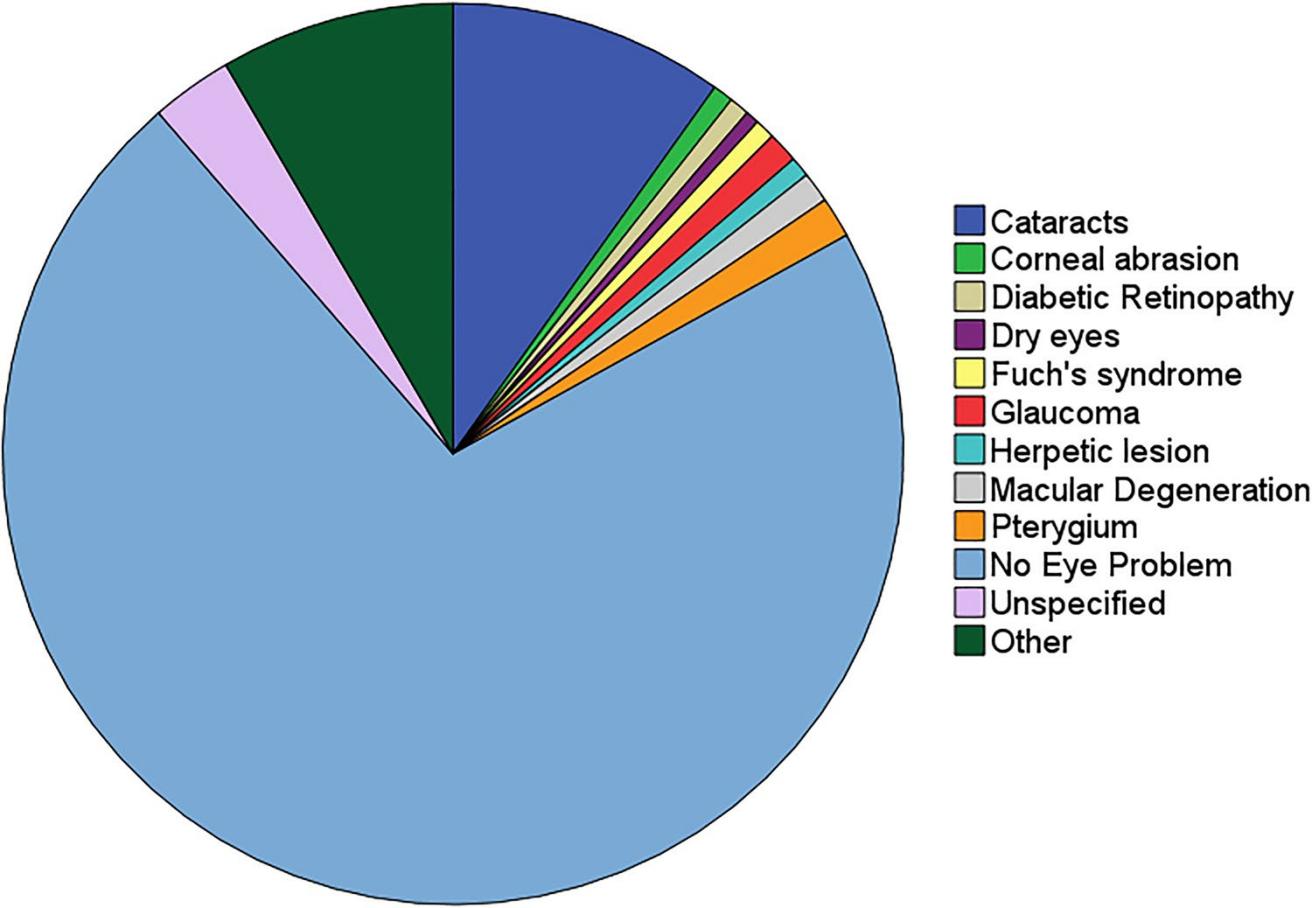
Pie chart showing prevalence data for reported “eye problems” in 259 sleep clinic patients. Cataracts (10%); corneal abrasion (1%); diabetic retinopathy (1%); dry eyes (1%); Fuch’s syndrome (1%); glaucoma (1%); herpetic lesion (1%); macular degeneration (1%); pterygium (surfer’s eye, 2%); no eye problem (76%); unspecified (3%); other (9%). Note: (1) does not include reports of long/short sightedness or use of spectacle/contact lenses; (2) total > 100% because some individuals reported more than one “eye problem”

Table 1A–C show group anthropometric, medical history, questionnaire, and PSG data. Figure 1 shows prevalence data for patient reported “eye problems.” Most patients (76%) reported “no eye problems,” while the most common specific condition nominated was “cataracts” (10%).

**Table 1:** Group demographics and sleep-disordered breathing status. Anthropometric, demographic, medical history, “eye problems” questionnaire, and polysomnography data presented as mean ± SD (A), frequency (B), or median (IQR) (C) and where appropriate, plus range, or percentage of total

A)	*N*	Mean ± SD	Range
Age (years)	396	56.1 ± 11.0	22–79
BMI (kg/m^2^)	393	32.2 ± 7.4	17.1–71.4
Systolic BP (mmHg)	389	129 ± 16	90–176
Diastolic BP (mmHg)	389	78 ± 9.0	51–110
B)	*N*	Frequency	Percentage
Gender (male)	396	226	57.1
Ethnicity (Caucasian)	396	240	60.6
Hypertension History	396	177	44.7
Hypercholesterolemia History	396	176	44.4
Diabetic History	396	29	7.3
Smoking history (including 20 current smokers)	396	177	44.7
Reported Eye Problems	259	70	27.0
C)	*N*	Median (IQ range)	Range
AHI (events/h)	385	12.6 (4.4–27.4)	0.0–103.9
RDI (events/h)	385	24.8 (14.2–42.4)	1.1–104.2
AI (events/h)	385	27.9 (18.9–41.1)	5.9–100.5
ODI 3% (events/h)	385	4.1 (1.1–10.0)	0.0–73.0
SpO_2_ < 90% (% TST)	385	0.6 (0.0–3.2)	0.0–99.6

*BMI* body mass index, *BP* blood pressure, *AHI* apnea–hypopnea index, *RDI* respiratory disturbance index, *AI* arousal index, *ODI 3%* oxygen desaturation (3%) index, *SpO*_*2*_ < *90%* percent of sleep time with oxygen saturation < 90

**Fig. 2:**
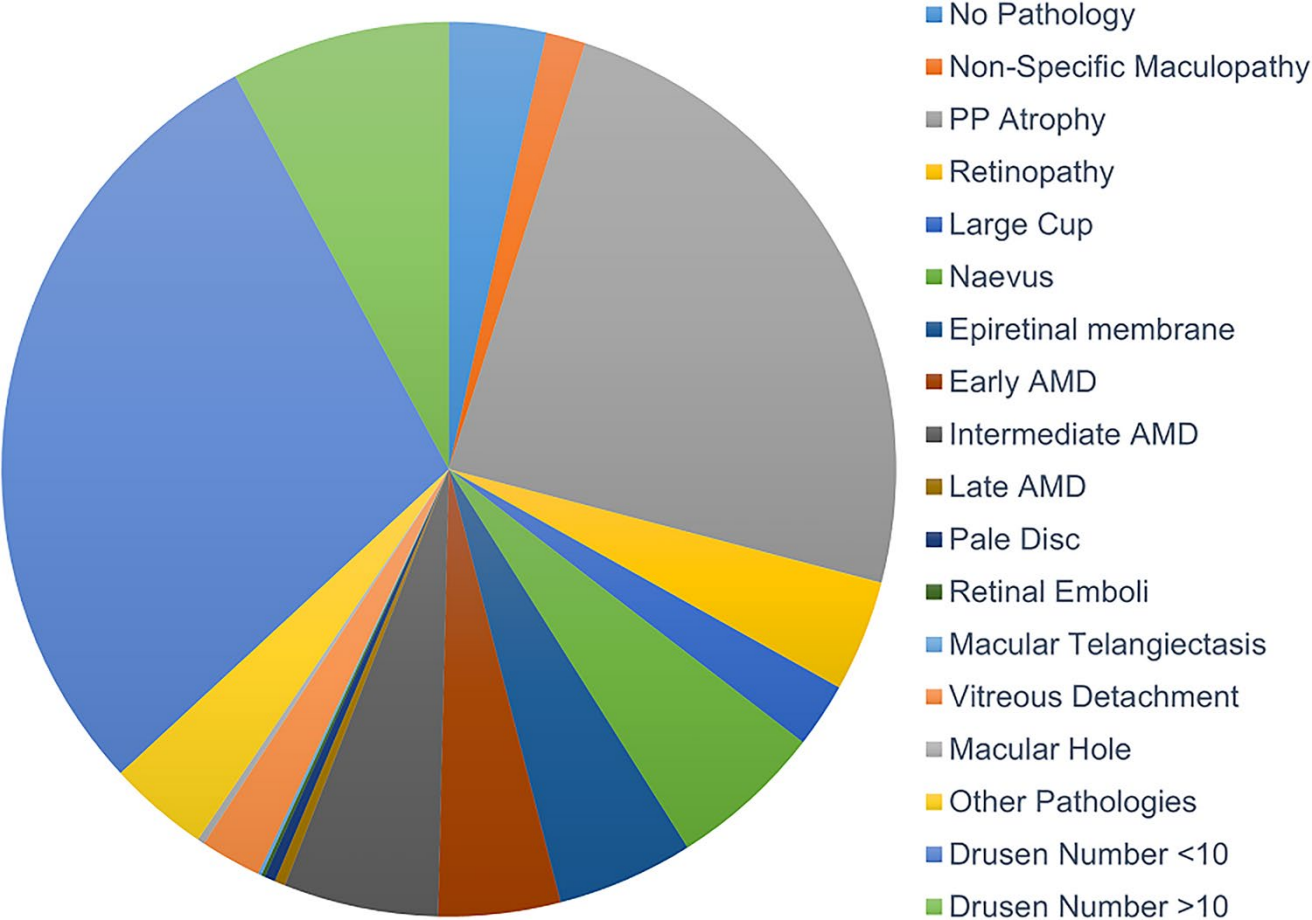
Pie chart showing prevalence data for retinal abnormalities identified by retinal photography in 396 sleep clinic patients. No pathology (7%); non-specific maculopathy (3%); peripapillary atrophy (PPA, 47%); retinopathy (8%); large cup (5%); naevus (11%); epiretinal membrane (10%; consisting of epiretinal membrane only (1%), surface wrinkling (3%) and cellophane reflex (8%); early age-related macular degeneration (AMD, 9%); intermediate AMD (11%); late AMD (1%); pale disc (1%); retinal emboli (0.3%); macular telangiectasis (0.3%); viterous detachment (4%); macular hole (1%); other pathologies (7%); drusen number < 10 (56%); drusen number ≥ 10 (15%)

Figure 2 reports prevalence data for retinal-photograph-identified abnormalities. The vast majority of patients (93%) were classified as having at least one retinal abnormality present in at least one eye. Only 7% of patients were identified as having no abnormal findings. A wide range of abnormalities was identified; the most common were drusen < 10 lesions (56%), drusen ≥ 10 lesions (15%), peripapillary atrophy (PPA; 47%), naevus (11%), epiretinal membrane (10%), and retinopathy (8%). AMD was identified as early in 9% of patients, intermediate in 11%, and late in 1%. Although a pale disc was noted in 1% of cases, NAION was not clinically reported for any patient.

Results for the logistic regression models for the most commonly encountered, and potentially clinically significant, abnormalities identified are shown in Table 2 (PPA) and Table S1 in the Supplemental Material (Retinopathy, Drusen ≥ 10, all AMD, and Epiretinal Membrane). For non-SDB metrics, age was an identified significant risk factor for PPA (*P* < 0.001, odds ratio (OR) = 1.46 [1.19–1.78] per decade), drusen ≥ 10 (*P* = 0.001, OR = 1.68 [1.23–2.30] per decade), AMD (*P* < 0.001, OR 1.81 [1.38–2.38] per decade), and epiretinal membrane (*P* < 0.001, OR 2.54 [1.73–3.73] per decade). Diabetic history was an expected risk factor for retinopathy (*P* = 0.001, OR = 4.98 [1.91–12.98]).

**Table 2:** Logistic regression model for peripapillary atrophy

Variables	*B*	SE	Exp (*B*)	95% CI for Exp (*B*)	*P* value
Peripapillary atrophy					
Constant	− 2.259	0.588	0.104	-	0.000
Age (per decade, years)	0.378	0.102	1.46	1.195–1.784	0.000
Ln AHI + 1	-	-	-	-	0.145
Ln RDI	-	-	-	-	0.755
Ln AI	-	-	-	-	0.641
Ln ODI (≥ 3%) + 1	-	-	-	-	0.484
Ln SpO_2_ < 90% + 1	-	-	-	-	0.084
AHI severe (≥ 30 events/h)	-	-	-	-	0.209
AHI very severe (≥ 50 events/h)	1.008	0.377	2.741	1.310–5.736	0.007

*B* unstandardised coefficient, *SE* standard error, *Exp (B)* odds ratio, *CI* confidence interval, *Ln* natural logarithm, *AHI* apnea–hypopnea index, *RDI* respiratory disturbance index, *AI* arousal index, *ODI 3%* oxygen desaturation (3%) index, *SpO*_*2*_ < *90%* percent of sleep time with oxygen saturation < 90%

No continuous variable metric for SDB severity emerged as a significant risk factor for any tested retinal abnormality. For the categorical SDB variables, very severe SDB (AHI: ≥ 50 events/h) was a significant risk factor for PPA (*P* = 0.007, OR = 2.74 [1.31–5.73]).

## Discussion

The main findings from this cross-sectional study in a cohort of sleep clinic patients were (1) most patients reported no pre-existing “eye problems”; (2) most patients had a least one retinal abnormality identified from retinal photographs; (3) main identified retinal abnormalities included drusen, peripapillary atrophy, naevus, epiretinal membrane, AMD, and retinopathy; (4) age and diabetic history were identified as non-SBD-related risk factors for specific retinal abnormalities; (5) continuous SDB metrics were not identified as significant risk factors for any retinal abnormality; however, (6) patients with very severe levels of SDB (AHI ≥ 50 events/h) were nearly three times more likely to have PPA, which is a potential marker for glaucoma risk [15].

There are a number of important limitations to this study, including the inherent bias associated with a cohort recruited for a different research study (e.g., under-representation of diabetic patients because of original study exclusion criteria) and the lack of objective diagnostic criteria for glaucoma. The study group size is relatively small in comparison with large community-based studies but, nevertheless, constitutes one of the largest series yet reported for sleep clinic patients where SDB status has been accurately confirmed by in-laboratory PSG.

There have now been a number of reviews and meta-analyses published on the state of knowledge for associations between OSA and eye diseases. Recently, Mentek and co-workers [16] reviewed 88 publications and concluded that only the association with NAION is sufficiently well-documented. No cases of definite NAION were specifically identified in the present study. Zhao et al. [17] and Sun et al. [18] reviewed published studies using optical coherence tomography to quantify retinal nerve fiber layer thickness (RNFL; a biomarker for early glaucomatous pathology) in patients with OSA and proposed that more severe OSA was likely associated with reduced RNFL thickness. Overall, however, it has been emphasized [19, 20] that most publications in the field are limited in that they (1) consist of small sample size case series or case-controlled studies; (2) tend to provide inadequate control for potential confounders; (3) are likely subject to selection bias; and (4) apply variable diagnostic criteria with respect to both the eye disease and the OSA. Often, the severity of SDB is not considered at all or is inadequately quantified. In this context, one of the main advances provided by the present study is the gold-standard quantification of SDB status via in-laboratory PSG performed to international benchmark criteria.

There are few prospective studies. Recently, Pedrotti and co-workers [21] reported comprehensive prospective ocular examinations in 296 patients undergoing unattended portable PSG monitoring for the evaluation of SDB and concluded that while eye diseases (particularly eyelid disorders) were more prevalent than might be expected in the general population, only glaucoma was significantly related to measures of SDB severity.

### Questionnaire responses

In the present study when sleep clinic patients were asked if they experienced “Eye Problems” (exclusive of long/short sightedness/use of spectacle/contact lenses), the vast majority reported no specific eye conditions and there were remarkably few reports of eye diseases with suspected linkages to OSA. Notably, there were no reports of “floppy eyelid syndrome” or “NAION,” only two reports of diabetic retinopathy, and three (1%) reports of glaucoma.

These results provide an indicative insight into the range of conditions likely to be reported if an eye disease history question was to be included as part of clinical history taking in sleep clinic settings. However, given the responses encountered, use of such a general screening questionnaire seems unlikely to provide any clinically useful information on putative eye diseases that may be linked to SDB status. A more focused questionnaire, including specific eye conditions such as floppy eyelid syndrome, NAION, glaucoma, and diabetic retinopathy, might elicit more relevant information and contribute to the clinical assessment of eye disease in sleep clinic patients.

### Retinal photograph*y*

In stark contrast to the results from the patient questionnaire assessment, the retinal photograph assessment process identified at least one retinal abnormality in at least one eye in 93% of patients. Thus, in this sleep clinic cohort, very few individuals were considered to have no identifiable retinal abnormalities on retinal photography. This is a very different outcome to that reported by Zapata et al. [22] for an optometry-attendance-based retinal photography screening study of 50,384 optometry patients in Spain, where 75% of retinal photographs were considered “normal”^1^. Differences in age may in part contribute to this discrepancy, but Zapata et al. report only a 31.5% prevalence of abnormalities for patients greater than 50 years of age. This is only about a third of the rate detected in our cohort with a mean age of ~ 56 years.

### Peripapillary atrophy

**Fig. 3:**
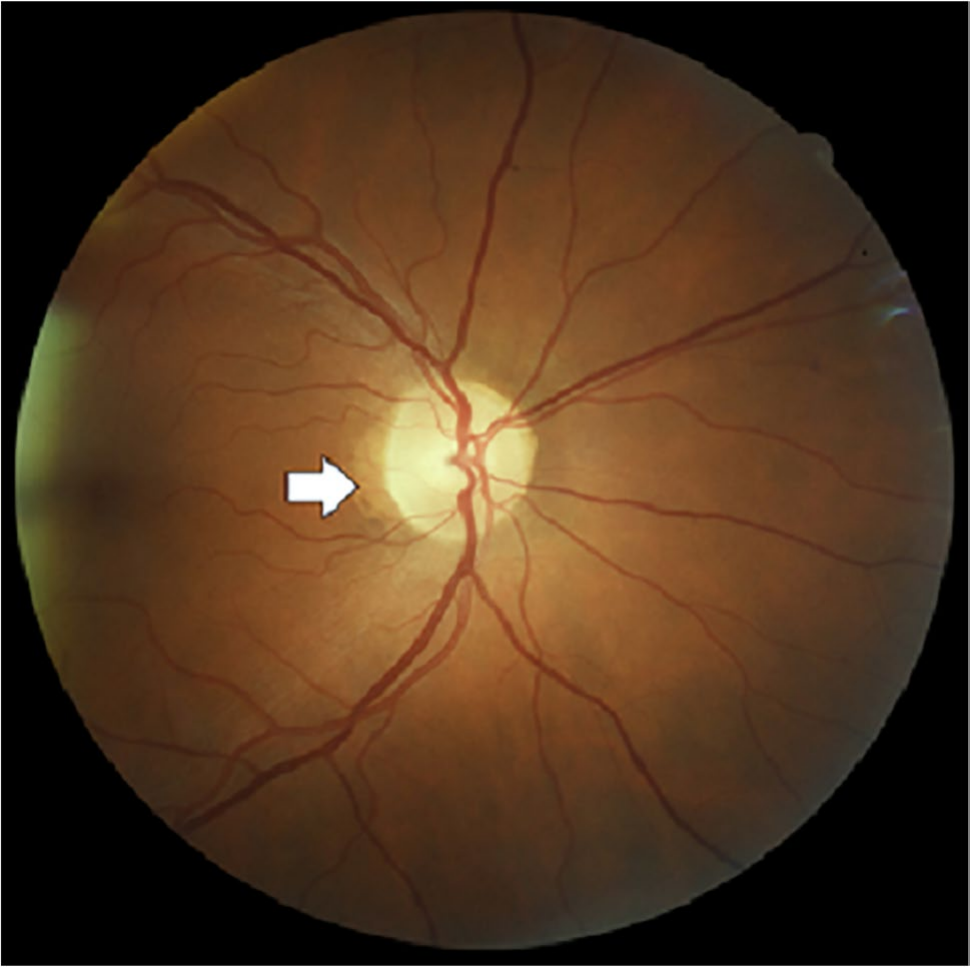
Example of peripapillary atrophy observed in a sleep clinic patient. Retinal photograph showing peripapillary atrophy (PPA; white arrow) in a 53-year-old male with very severe OSA (AHI: 81.3 events/h)

PPA is associated with chorioretinal thinning and pigment disruption surrounding the optic disc. It has been described in otherwise normal eyes, as well as in association with glaucoma [15]. It is weakly associated with age, with about a 0.2-mm increase in extent per decade of age [23], and is often classified according to its location: a central beta zone and a more peripheral alpha zone [24]. Figure 3 shows PPA in a 53-year-old male from our cohort with very severe OSA (AHI: 81.3 events/h).

The Rotterdam Study [24] found a “one or both eye” prevalence rate for alpha zone atrophy of 61.4% for women and 67.0% for men in the 55–64-year age group; corresponding rates for beta zone atrophy were 8.9% for women and 13.8% for men. Similar prevalence rates have been reported from the Beijing Eye Study [23]*.*

Investigators have suggested an association between PPA and glaucoma [15] with decreased peripapillary RNFL thickness considered to be a sign of progression in glaucoma [25]. Given the wide interest in the potential relationship between glaucoma and OSA [6], combined with the growing literature supporting thinning of the RNFL in the eyes of patients with OSA [26], our results, identifying very severe OSA as a significant risk factor for PPA, may add to evidence supporting a relationship between OSA severity and glaucoma.

### Diabetic retinopathy

Diabetic retinopathy is a leading cause of vision loss in the general population and is of particular interest in sleep clinic patients since the prevalence of diabetes may be high in these cohorts. Furthermore, OSA itself has been suggested as a risk factor for both the development and progression of diabetic retinopathy [10], although this conclusion is limited by a reliance on cross-sectional studies and inconsistent reporting of sleep study methodologies and OSA assessment criteria. However, one suggested mechanism for a link between OSA and diabetic retinopathy is nocturnal hypoxia, to which the diabetic retina is particularly vulnerable due to having the highest metabolic needs when asleep in the dark, and with reduced ability to increase retinal blood flow [27].

Interpretation of retinopathy prevalence in the present study cohort (8%) is limited because exclusion of diabetes from the majority of this cohort means that diabetes is likely under-represented in the study group as a whole. However, when SDB severity categorical variables were tested, severe SDB (AHI: ≥ 30 events/h) emerged as a borderline significant (*P* = 0.07) risk factor (see Supplemental Material Table S1).

### Drusen

Drusen are lipid deposits under the retina and occur naturally with age. They can be classified as “hard” (small, < 63um, distinct, can be calcified) or “soft” (larger, > 63um, in clusters with indistinct edges, contain liquefied material) [28]. Soft drusen can also be classified as distinct (uniform) or indistinct (fuzzy edges). Drusen are also classified by site, e.g., macular, optic disc, reticular (sub-retina). Large numbers of drusen have been linked to increased incidence of AMD [29] and accordingly, and in alignment with the AMD criteria of Ferris et al. 2013 [14], we classified drusen on the basis of the number present (< 10 versus ≥ 10).

There have been no previous studies of drusen in sleep clinic cohorts. In the present study, 56% of patients had < 10 drusen identified, while 15% had ≥ 10 drusen identified with age a significant risk factor for ≥ 10 drusen. SDB status, however, was not identified as a risk factor for drusen.

### Age-related macular degeneration

Age-related macular degeneration (AMD) is an acquired degeneration of the retina that causes significant central visual impairment in the late stages. It is a major cause of vision loss across the world. Its diagnosis, classification, epidemiology, and risk factors were recently reviewed by Mitchell et al. [30]. Age is the strongest risk factor.

Interest in the possible relationship between AMD and OSA has centered on the potential for AMD progression to accelerate in OSA patients [10] along with the suggestion that OSA may reduce the effectiveness of (anti-VEGF) therapy [11].

In the present study, AMD was identified as early in 9% of patients, intermediate in 11%, and late in 1%. Logistic regression analysis confirmed age as the major risk factor for AMD, but failed to identify any relationship with SDB status. However, this does not necessarily obviate any effect on AMD progression (not evaluated in the current cross-sectional study).

## Conclusion

Findings in the present study suggest that deploying retinal photography as a screening tool in sleep clinics will identify a range of undiagnosed retinal pathological conditions of varying health significance, most of which will be related to the age and co-morbidity status (e.g., diabetes) of patients typically referred to such clinics, and not related to SDB severity.

## Supplementary Information

Below is the link to the electronic supplementary material.

Supplementary file1 (DOCX 22 KB)

## Data Availability

The datasets generated during and/or analyzed during the current study are available from the corresponding author on reasonable request.

## Abbreviations

AHI: Apnoea/hypopnea index
AI: Arousal index
Anti-VEGF: Anti-vascular endothelial growth factor
ARMD: Age-related macular degeneration
BMI: Body mass index
CI: Confidence intervals
CPAP: Continuous positive airway pressure
NAION: Non-artertic anterior ischemic optic neuropathy
ODI: Oxygen saturation index
OR: Odds ratio
OSA: Obstructive sleep apnea
PPA: Peripapillary atrophy
PSG: Polysomnography
RDI: Respiratory disturbance index
RNFL: Retinal nerve fiber layer
SaO_2_ < 90%: Percent of sleep time with oxygen saturation < 90%
SD: Standard deviation
SDB: Sleep-disordered breathing
SE: Standard error
SWR: Surface wrinkling retinopathy
TST: Total sleep time
WHR: Waist-hip ratio
