# Supplementary material for: Retinal abnormalities, although relatively common in sleep clinic patients referred for polysomnography, are largely unrelated to sleep-disordered breathing
Source: Sleep Breath. 2022 Jul 8;27(3):861–8. doi: 10.1007/s11325-022-02679-y (PMC10227130; doi:10.1007/s11325-022-02679-y)
Supplement: Supplementary file 1 — Supplementary file1 (DOCX 22 KB) [file 11325_2022_2679_MOESM1_ESM.docx]

**Supplementary Material**

**Table S1 - Logistic regression models for specific retinal abnormalities.**

| **Variables** | **B** | **SE** | **Exp (B)** | **95% CI for Exp (B)** | **P value** |
| --- | --- | --- | --- | --- | --- |
| **Retinopathy** | | | | | |
| **Constant** | -2.654 | 0.216 | 0.07 | - | 0 |
| **Age (per decade, years)** | - | - | - | - | 0.121 |
| **Diabetic History** | 1.605 | 0.489 | 4.976 | 1.907 – 12.983 | 0.001 |
| **Ln AHI + 1** | - | - | - | - | 0.256 |
| **Ln RDI** | - | - | - | - | 0.449 |
| **Ln AI** | - | - | - | - | 0.653 |
| **Ln ODI (≥ 3%) + 1** | - | - | - | - | 0.528 |
| **Ln SaO_2_ < 90% + 1** | - | - | - | - | 0.573 |
| **AHI severe (≥ 30 events/hr)** | 0.744 | 0.410 | 2.104 | 0.942 – 4.701 | 0.070 |
| **AHI very severe ≥ 50 events/hr)** | - | - | - | - | 0.936 |
| **Drusen Number ≥ 10** | | | | | |
| **Constant** | -4.280 | 0.958 | 0.014 | - | 0 |
| **Age (per decade, years)** | 0.519 | 0.160 | 1.680 | 1.228 – 2.300 | 0.001 |
| **Smoking History** | - | - | - | - | 0.283 |
| **Ln AHI + 1** | - | - | - | - | 0.873 |
| **Ln RDI** | - | - | - | - | 0.856 |
| **Ln AI** | - | - | - | - | 0.832 |
| **Ln ODI (≥ 3%) + 1** | - | - | - | - | 0.640 |
| **Ln SaO_2_ < 90% + 1** | - | - | - | - | 0.341 |
| **AHI severe (≥ 30 events/hr)** | - | - | - | - | 0.924 |
| **AHI very severe ≥ 50 events/hr)** | - | - | - | - | 0.646 |
| **Age-Related Macular Degeneration** | | | | | |
| **Constant** | -4.913 | 0.847 | 0.007 | - | 0.000 |
| **Age (per decade, years)** | 0.593 | 0.139 | 1.810 | 1.378 – 2.378 | 0.000 |
| **Smoking History** | - | - | - | - | 0.606 |
| **Hypertension History** | - | - | - | - | 0.595 |
| **Ln AHI + 1** | - | - | - | - | 0.753 |
| **Ln RDI** | - | - | - | - | 0.252 |
| **Ln AI** | - | - | - | - | 0.495 |
| **Ln ODI (≥ 3%) + 1** | - | - | - | - | 0.398 |
| **Ln SaO_2_ < 90% + 1** | - | - | - | - | 0.138 |
| **AHI severe (≥ 30 events/hr)** | - | - | - | - | 0.691 |
| **AHI very severe ≥ 50 events/hr)** | - | - | - | - | 0.666 |
| **Epiretinal Membrane Cluster** | | | | | |
| **Constant** | -7.804 | 1.250 | 0 | - | 0.000 |
| **Age (per decade, years)** | 0.933 | 0.195 | 2.543 | 1.735 – 3.729 | 0.000 |
| **Ethnicity** | - | - | - | - | 0.757 |
| **Ln AHI + 1** | - | - | - | - | 0.510 |
| **Ln RDI** | - | - | - | - | 0.491 |
| **Ln AI** | - | - | - | - | 0.520 |
| **Ln ODI (≥ 3%) + 1** | - | - | - | - | 0.656 |
| **Ln SaO_2_ < 90% + 1** | - | - | - | - | 0.709 |
| **AHI severe (≥ 30 events/hr)** | - | - | - | - | 0.276 |
| **AHI very severe ≥ 50 events/hr)** | - | - | - | - | 0.772 |

SE = Standard error, CI = confidence interval, AHI = apnea-hypopnea index, RDI = respiratory disturbance index, AI = arousal index, ODI > 3% = oxygen desaturation (> 3%) index, SaO_2_ <90% = percent of sleep time with oxygen saturation <90%.
